# Intelligent skin disease prediction system using transfer learning and explainable artificial intelligence

**DOI:** 10.1038/s41598-024-83966-4

**Published:** 2025-01-11

**Authors:** Sagheer Abbas, Fahad Ahmed, Wasim Ahmad Khan, Munir Ahmad, Muhammad Adnan Khan, Taher M. Ghazal

**Affiliations:** 1 Department of Computer Science, Prince Mohammad Bin Fahd University, 34754 Al-Khobar, Dhahran, KSA Saudi Arabia; 2 School of Computer Science, National College of Business Administration and Economics, Lahore, 54000 Pakistan; 3 Department of Computer Science, Baba Guru Nanak University, Nankana Sahib, 39100 Pakistan; 4 College of Informatics, Korea University, Seoul, 02841 Republic of Korea; 5 Department of Software, Faculty of Artificial Intelligence and Software, Gachon University, Seongnam-si, 13120 Republic of Korea; 6 Research Innovation and Entrepreneurship Unit, University of Buraimi, 512, Buraimi, Oman; 7 Center for Cyber Security, Faculty of Information Science and Technology, Universiti Kebangsaan Malaysia (UKM), Bangi, 43600 Selangor Malaysia

**Keywords:** Artificial intelligence (AI), Machine learning (ML), Deep learning (DL), Transfer learning (TL), VGG16, Chickenpox, Measles, Monkeypox, Explainable artificial intelligence (XAI), And layer-wise relevance propagation (LRP), Skin cancer, Computational science, Computer science, Software

## Abstract

Skin diseases impact millions of people around the world and pose a severe risk to public health. These diseases have a wide range of effects on the skin’s structure, functionality, and appearance. Identifying and predicting skin diseases are laborious processes that require a complete physical examination, a review of the patient’s medical history, and proper laboratory diagnostic testing. Additionally, it necessitates a significant number of histological and clinical characteristics for examination and subsequent treatment. As a disease’s complexity and quantity of features grow, identifying and predicting it becomes more challenging. This research proposes a deep learning (DL) model utilizing transfer learning (TL) to quickly identify skin diseases like chickenpox, measles, and monkeypox. A pre-trained VGG16 is used for transfer learning. The VGG16 can identify and predict diseases more quickly by learning symptom patterns. Images of the skin from the four classes of chickenpox, measles, monkeypox, and normal are included in the dataset. The dataset is separated into training and testing. The experimental results performed on the dataset demonstrate that the VGG16 model can identify and predict skin diseases with 93.29% testing accuracy. However, the VGG16 model does not explain why and how the system operates because deep learning models are black boxes. Deep learning models’ opacity stands in the way of their widespread application in the healthcare sector. In order to make this a valuable system for the health sector, this article employs layer-wise relevance propagation (LRP) to determine the relevance scores of each input. The identified symptoms provide valuable insights that could support timely diagnosis and treatment decisions for skin diseases.

## Introduction

A severe worldwide health concern that affects a large number of people is skin disease. Due to their physical and psychological impacts on individuals, skin diseases are a serious and concerning problem in societies^1^. Early-level detection of the kind of disease is vital in determining the appropriate treatments that can be carried out.

Varicella zoster virus (VZV) causes chickenpox^2^. VZV belongs to the herpes virus family^3^. The majority of affected children are between 6 months and 7 years old^4^. Chickenpox, the highly contagious disease that affects children, is now being reported to affect significant portions of adults^5^. The main symptom is an itchy, red rash that frequently turns into blisters and progresses to flu-like symptoms. It spreads through contact with sick individuals, contaminated objects, and airborne droplets brought on by coughing or sneezing. Additionally, chickenpox can be dangerous and even fatal, particularly in pregnant women^6^.

High fever, cough, coryza, conjunctivitis, and a morbilliform rash are all symptoms of the highly contagious and possibly fatal airborne measles^7^. Measles is a fatal viral disease carried on by a paramyxoviridae family RNA virus^8^. Despite being seen as a childhood disease, measles can affect people of all ages. A single measles case has been shown to result in 12–18 secondary infections in an otherwise healthy group^9^.

With the coronavirus disease (COVID-19) pandemic still raging, just as people are beginning to adjust to the ‘new normal,’ the monkeypox virus is wreaking havoc on the world. Healthcare professionals around the globe are progressively concerned regarding the most recent monkeypox outbreak. The Democratic Republic of the Congo (previously Zaire) received the first human monkeypox infection report in 1970^10^. The monkeypox virus is a zoonotic pathogen that renders a rash akin to that of smallpox^11^. According to research, the Poxviridae family, of which the monkeypox virus is a member, was first spread from animals to people^12^. It can be spread by respiratory droplets, animal bites, mouth, nose, or eye mucus, or by direct contact with another person^13^.

Compared to COVID-19, monkeypox is not as contagious, although the number of cases is still rising. In 1990, there were just 50 cases of monkeypox recorded in West and Central Africa^14^. However, 5,000 cases were reported in 2020. Despite the consensus that monkeypox only happened in Africa, cases of the virus were documented in 2022 in several non-African countries in Europe and the US^15^. Because of this, people are progressively growing more fearful and nervous, which typically shows in their thoughts on social media. Scientists attribute the current outbreak of monkeypox in humans, thought to be occurring on a global scale, to either a change in the essential characteristics of the monkeypox virus or adjustments in the human lifestyle^16^.

Lately, there has been a significant growth in the amount and quality of research in many areas using machine learning (ML) to classify skin lesions^17^. However, to improve ML models, effective feature extraction techniques are required. Traditional ML classifiers have the disadvantage that one must create complex hypotheses independently, whereas deep neural networks generate them automatically, making them an effective tool for learning non-linear correlations^18^. Due to their increased success in processing enormous amounts of data and the capability to extract hidden valuable knowledge from data, deep learning (DL) approaches have historically diverged from traditional ML techniques^19^. The DL has been successfully utilized in numerous areas, like lesion detection^20–22^, classification^23–29^, and segmentation of medical images^30,31^.

Additionally, hybrid approaches such as Adaptive Neuro-Fuzzy Inference Systems (ANFIS) have been applied to tasks like sentiment analysis^32^. Convolutional neural networks (CNNs) are generally utilized in DL to learn features automatically and use that information for classification. CNNs are a class of neural networks typically used on data where the input has another tensor structure, such as an image. They are specifically designed to capture the inherent structure of images and visual data through a sequence of Modules with interconnected Nodes, which automatically generate spatial hierarchies of features, making them ideal for image recognition, image segmentation, object detection, and image classification problems.

**Fig. 1:**
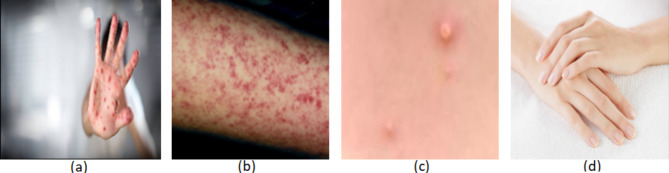
Skin images of four classes: (**a**) Chickenpox; (**b**) Measles; (**c**) Monkeypox; (**d**) Normal.

Given that DL is the state-of-the-art for analyzing medical images^33,34^, it is not surprising that medical professionals have expressed their concerns regarding the technology’s “black box”^35^. The need for more transparency and fairness in DL models is a notable issue, specifically in critical areas such as healthcare, where trust and understanding of artificial intelligence (AI) predictions are vital. To tackle this issue, explainable artificial intelligence (XAI) has emerged as a powerful approach, allowing AI models to produce results that people can understand and trust. This article proposes a model for identifying and predicting skin diseases using transfer learning (TL) empowered with XAI. The primary objective is to address the shortcomings of current methods, such as low accuracy and a lack of explainability, by creating a model that is more precise and easier to interpret. The expected result is a more accurate and interpretable AI model, addressing the common issues of low performance and lack of transparency in current healthcare applications. TL employs pre-trained CNNs, utilizing learned features to facilitate the adaptation of models to new tasks, hence minimizing the training time and data needs. XAI refers to AI approaches that produce results humans can interpret or comprehend. Figure 1 shows chickenpox, measles, monkeypox, and normal skin images.

The remainder of the article is divided as follows: Sect. "Literature review" discusses the literature review, Sect. "Materials and methods" discusses materials and methods, Sect. "Simulation and results" offers the simulation and results, Sect. "Practical and managerial implications" gives practical and managerial implications, and finally, Sect. "Conclusion and future work" provides the conclusion and future work.

## Literature review

Skin disease is one of the most widespread diseases among people. Skin diseases range from superficial acne to severe conditions like squamous cell carcinoma. It affects people of all cultures, regions, and age groups. In the last decade, skin and subcutaneous diseases have been the fourth leading reason for the worldwide burden of non-fatal diseases^36^. Despite involving most of the population at a time, they do not get much importance from a public health point of view. The Global Burden of Disease Study 2017 reported that years of life lost due to skin disease are between 30 and 40 months for an adult^37^. Recognizing disease is crucial for choosing the most appropriate treatment and preventing its spread.

The pertinent data on the age and gender of chickenpox patients reveals that there is no real difference in the gender of individuals who are affected. Children, however, are the age group with the highest prevalence of chickenpox, mainly because they exhibit group social features and are more likely to spread the disease to those around them. Currently, vaccination is the only method of mass prevention that has been scientifically shown to be both practical and economical. In the US, a varicella vaccine surveillance study found that 13% of children between the ages of 5 and 10 are susceptible to the disease. Similarly, studies conducted in the UK show that approximately 40% of kids aged 1 to 9 are vulnerable to infection, but less than 10% of kids over 15 are^38^. The disease burden has significantly decreased in developed nations, and most developed nations have included the varicella vaccine as part of their standard immunization plan^39^. Roy et al.^40^ used various segmentation approaches to identify skin diseases like chickenpox, candidiasis, cellulitis, and acne.

A severe, contagious viral disease is measles. Before the measles vaccine was created in 1963, massive measles epidemics happened every two to three years, resulting in an estimated 2.6 million measles fatalities yearly. However, between 2000 and 2016, there was an 84% decline in measles mortality as the vaccine became more readily available^41^. Vaccination is a reasonably effective way to avoid measles.

Until 1958, reports of smallpox-like diseases in monkeys were sporadic, and monkeypox was relatively unknown^42^. Intense smallpox surveillance in the Democratic Republic of the Congo, where smallpox was considered eliminated, led to the identification of the first human case of monkeypox in 1970. The patient, a nine-month-old boy with hemorrhagic monkeypox, survived the infection^43^. Before 2003, only African countries had recorded human cases of monkeypox^44^. A multi-state zoonotic outbreak in the USA that lasted from May to June 2003 was the cause of the first human cases of monkeypox outside of Africa^45^. Monkeypox outbreaks have been documented in several nations, primarily in Europe, since the beginning of May 2022, although the monkeypox virus is not prevalent in those regions^46^.

The ‘Monkeypox Skin Lesion Dataset (MSLD)’ was developed by Ali et al.^47^ and includes skin lesion images of chickenpox, measles, and monkeypox, with the majority of images sourced from publicly accessible case reports, blogs, and news websites. The sample size is expanded through data augmentation, and a 3-fold cross-validation experiment is set up. Different pre-trained DL models, including VGG16, ResNet50, and InceptionV3, are used to classify monkeypox and other diseases. Additionally, an ensemble of the three models is created. VGG16, ResNet50, InceptionV3, and ensemble achieved accuracies of 81.48 (± 6.87%), 82.96 (± 4.57%), 74.07 (± 3.78%), and 79.26(± 1.05%), respectively. Burak Gülmez^48^ developed a hybrid DL model, “MonkeypoxHybridNet,” by combining three pre-trained models—ResNet50, VGG19, and InceptionV3. This model was trained on the “Monkeypox2022” dataset and attained an accuracy of 84.2%.

Irmak et al.^49^ utilized pre-trained DL architectures to detect monkeypox skin lesions. This study’s classification used the monkeypox skin image dataset, which was open-sourced in 2022. The dataset contains four classes: chickenpox, measles, monkeypox, and normal. Pre-trained DL architectures, MobileNetV2, VGG16, and VGG19, were trained. MobileNetV2 had the best performance result, with an accuracy of 91.38% compared to VGG16 and VGG19.

Singh and Songare^50^ applied the DL models InceptionV3, GoogLeNet, ResNet50, and VGG16 to a two-class dataset containing normal and monkeypox classes and discovered that the GoogLeNet model had the highest accuracy at 88.27%. Sharma et al.^51^ developed a custom ResNet-18-based model for detecting monkeypox, measles, and chickenpox and compared it to several other models. Their model’s accuracy was 84.59%. Using Darknet 19 and Improved Darknet 19, Sethy et al.^52^ suggested a novel technique for the early diagnosis of monkeypox in their study. The research dataset included samples of skin diseases like chickenpox, measles, monkeypox, and normal cases. Darknet 19 and Improved Darknet 19 models were reported to have attained accuracies of 81.4% and 85.49%, respectively.

Uysal^53^ created a hybrid AI system capable of detecting monkeypox in skin images. This dataset contains four classes. In the original dataset, the data distribution of the classes is unbalanced. Several data augmentation and data preprocessing techniques were employed to rectify this disparity. The test accuracy of the hybrid AI system devised and suggested for monkeypox detection was 87%. Ariansyah et al.^54^ suggested a CNN and VGG16-based classification methodology to identify the symptoms of monkeypox and measles. The image dataset used in this proposed methodology contains the classes of monkeypox, measles, and normal. VGG16 achieves a high accuracy of 83.33% as compared to CNN.

Kundu et al.^55^ proposed an ML and DL classification methodology for monkeypox prediction. Monkeypox and others (which include chickenpox or measles) are two categories of skin lesions that are included in the dataset. Support vector machine (SVM) and k-nearest neighbor (KNN) were utilized as ML algorithms, while vision transformer (ViT) and RestNet50 were utilized as DL algorithms. Among the ML models, the KNN attains the best accuracy of 84%. However, with an accuracy of 93%, the ViT acts better than the other models.

Aqsa Akram et al.^56^ introduced “SkinMarkNet,” a novel technique for classifying monkeypox lesions utilizing an ensemble of three TL models—Inception, Xception, and ResNet. The study addresses the scarcity of annotated data by using data augmentation techniques, which enhance the training dataset and improve the model’s performance. The dataset, consisting of diverse skin lesion images from the Kaggle repository, was used to train the model. “SkinMarkNet” achieved a high classification accuracy of 90.615%, outperforming traditional ML and DL methods. The research shows the prospect of combining advanced DL models and data augmentation to enhance the automated diagnosis of monkeypox, contributing to more effective public health responses.

**Table 1:** Limitations of related work.

Author	Image based Dataset	Technique	Outcomes	Limitations
Ali et al.^47^	Chickenpox, measles, and monkeypox	VGG16, ResNet50, InceptionV3, and Ensemble	Achieved accuracies of 81.48 (± 6.87%), 82.96 (± 4.57%), 74.07 (± 3.78%), and 79.26(± 1.05%).	(i) Low accuracy (ii) No use of explainable artificial intelligence
Burak Gülmez^48^	Chickenpox, measles, monkeypox, and normal	Ensemble approach	Achieved an accuracy of 84.2%.	(i) Low accuracy (ii) Small dataset (iii) No use of data augmentation (iv) No use of explainable artificial intelligence
Irmak et al.^49^	Chickenpox, measles, monkeypox, and normal	MobileNetV2, VGG16, and VGG19	Achieved accuracies of 91.38%, 83.62%, and 78.45%.	(i) Low accuracy (ii) Small dataset (iii) No use of data augmentation (iv) No use of explainable artificial intelligence
Singh and Songare^50^	Monkeypox and normal	VGG-16, ResNet50, InceptionV3, and GoogLeNet	Achieved accuracies of 83.85%, 85.38%, 86.37%, and 88.27%.	(i) Low accuracy (ii) Less classes (iii) No use of explainable artificial intelligence
Sharma et al.^51^	Monkeypox, measles, and chickenpox	ResNet18-based model	Achieved an accuracy of 84.59%.	(i) Low accuracy (ii) Small dataset (iii) No use of explainable artificial intelligence
Sethy et al.^52^	Chickenpox, measles, monkeypox, and normal	Darknet 19 and Improved Darknet 19	Achieved accuracies of 81.4% and 85.49%.	(i) Low accuracy (ii) Small dataset (iii) No use of data augmentation (iv) No use of explainable artificial intelligence
Uysal^53^	Chickenpox, measles, monkeypox, and normal	CNN-LSTM hybrid model	Achieved an accuracy of 87%.	(i) Low accuracy (ii) No use of explainable artificial intelligence
Ariansyah et al.^54^	Monkeypox, measles, and normal	CNN and VGG16	Achieved accuracies of 64.52% and 83.33%.	(i) Low accuracy (ii) Small dataset (iii) No use of data augmentation (iv) No use of explainable artificial intelligence
Kundu et al.^55^	Chickenpox, measles, and monkeypox	SVM, KNN, RestNet50, and ViT	Achieved accuracies of 65%, 84%, 91%, and 93%.	(i) Low accuracy (ii) No use of explainable artificial intelligence
Aqsa Akram et al.^56^	Chickenpox, measles, monkeypox, and normal	SkinMarkNet	Achieved an accuracy of 90.615%.	(i) Low accuracy (ii) No use of explainable artificial intelligence

There are a few prominent limitations regarding the previous research, as given in Table 1.

1. There is area for improvement in the overall accuracy of previous literature^47–56^
2. No use of explainable artificial intelligence^47–56^

The noteworthy contributions of this proposed article are as follows:

1. Skin diseases have been identified and predicted using this proposed model.
2. The proposed model classifies chickenpox, measles, monkeypox, and normal skin images into their respective classes.
3. The performance metrics for the proposed model demonstrate encouraging outcomes, including accuracy, misclassification rate, precision, specificity, sensitivity, false negative rate (FNR), false positive rate (FPR), and F1 score.
4. The main contributions of this proposed model are improved accuracy relative to previous works and the incorporation of the XAI approach layer-wise relevance propagation (LRP) to explain the decision-making process of DL predictions better.

## Materials and methods

**Fig. 2:**
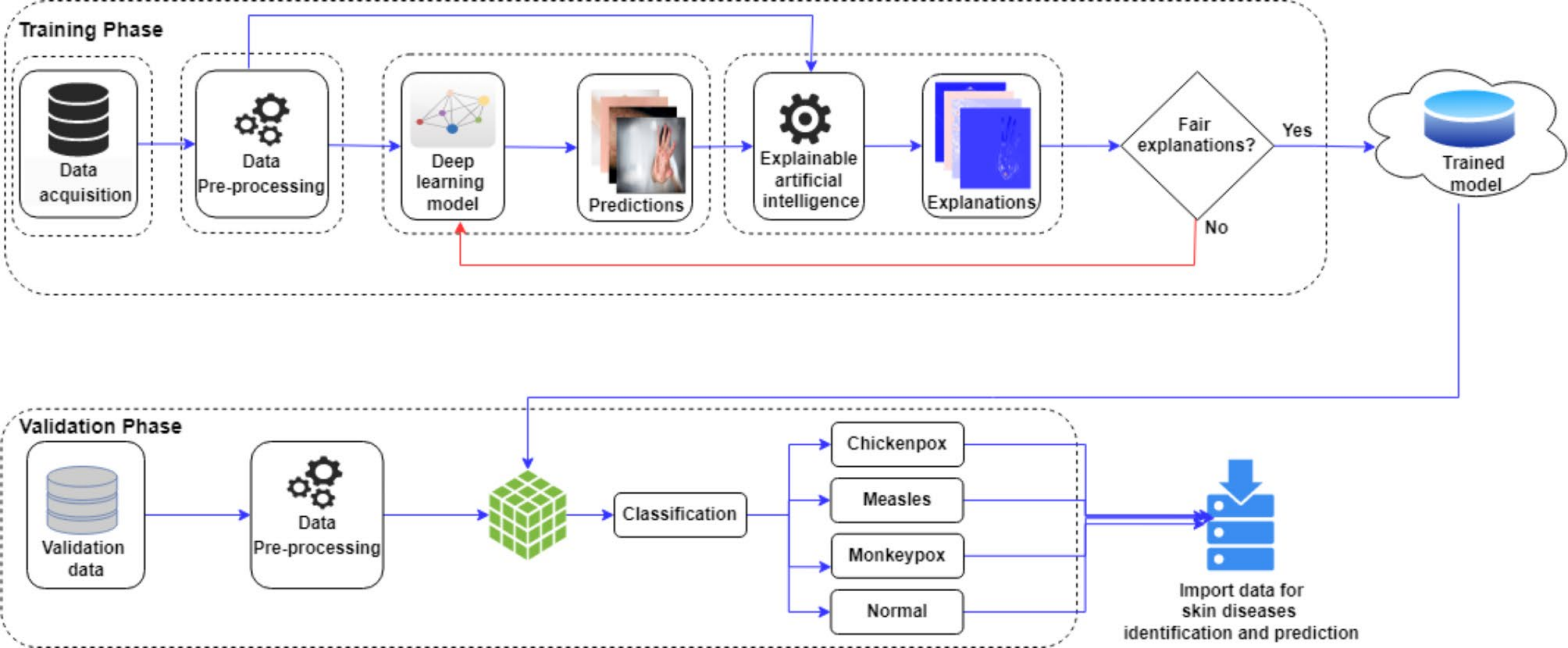
Framework of the proposed model.

Adopting AI techniques may be beneficial for routine screening for the early identification of prevalent skin diseases. Figure 2 displays the framework of the proposed model. The proposed model has five layers and two phases: training and validation.

In the training phase, layer 1 describes obtaining raw skin disease data from the open source. In layer 2, raw data is pre-processed according to the DL model. In data pre-processing, raw images acquired from open source are then converted into processed images with the RGB dimensions of 224 × 224 × 3. 224 × 224 denotes the length and width, while 3 denotes the channel count. After pre-processing, the data is randomly separated into training and testing sets for each of the four classes. For every class, 80% of the data is used for training and 20% for testing, keeping the overall dataset in the same 80:20 proportion. The pre-trained VGG16 model is imported and modified for the DL model. Layer 3 defines the predictions made by the DL model. These predictions may be perfect for decision-making, but they must explain how the DL model reached this decision. This prediction model of DL is known as the black box. To bring fairness to the decision-making process, the DL model is entangled with explainable artificial intelligence in layer 4. XAI method attempts to address the issue of opaqueness in DL models by explaining decisions based on comparisons between a model’s predictions and pre-processed data. If these explanations show any biases or inconsistencies, the model will be retrained to perform better in fairness and accuracy. When the explanations are good enough, this model will be saved on the cloud for future use. It will ensure a reliable and defensible model as this iterative process is performed.

During the validation step, which is the fifth layer of the model, the trained model is imported from the cloud to verify the pre-processed data obtained from different sources. The proposed model predicts and identifies the skin image data into four distinct classes, each with an explanation. After successfully identifying and predicting skin diseases, data is imported for future use, as shown in Fig. 2.

### Skin images dataset

The study used an open-source dataset of skin diseases^57^. Chickenpox (107), measles (91), monkeypox (279), and normal (293) are the four classes, and a total of 770 images are present. Table 2 describes classes and the number of image samples after the data augmentation.

**Table 2:** Dataset parameters.

Classes	No. of Image Samples
Chickenpox	321
Measles	273
Monkeypox	837
Normal	879
Total	2310

### Transfer learning

TL is a DL approach that uses pre-trained networks for various applications that can be used in the same domain or over different domains. The idea behind TL is to use models already trained on large and representative datasets rather than building a new CNN from scratch for each new task. With such pre-training, the first few layers of the network are being trained to extract low-level features like edges and colors, which generalize across multiple problems. It makes the learned model available for use in other applications. Based on the problem, one can fine-tune later layers of a network to adapt according to specific needs with a few more iterations trained, not the whole training again. VGG16 is utilized in this study to identify and predict skin diseases. VGG16, a deep CNN architecture with 16 layers, was constructed by Simonyan and Zisserman of the University of Oxford^58^. It has 16 layers that have learnable weight parameters.

#### VGG16

**Fig. 3:**
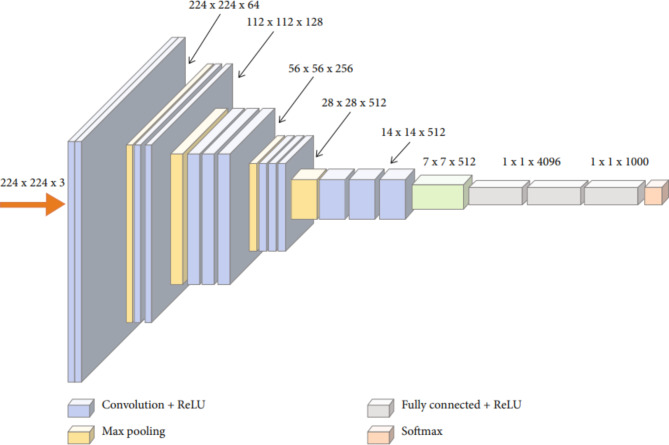
VGG16 original architecture^59^.

The 16 layers in this proposed VGG16 architecture include 3 fully connected layers, a max-pooling layer, and 13 convolutional layers, as displayed in Fig. 3. The input layer’s images are 224 × 224 × 3 in size, and the classification layer is the last.

**Fig. 4:**
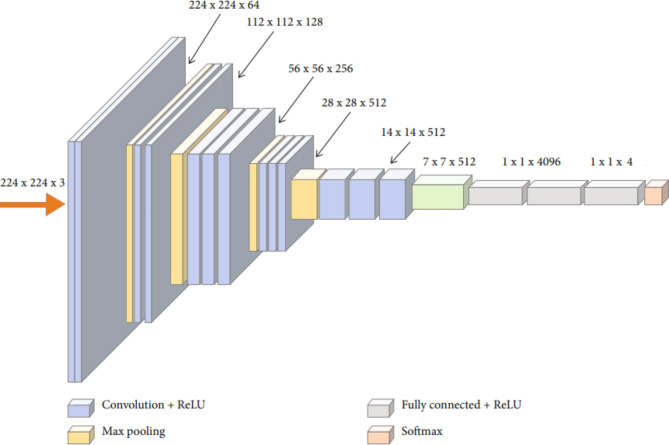
Modified VGG16 architecture.

The VGG16 model is employed in the current study to classify four classes of skin images. Figure 3 shows the VGG16’s original architecture before modification. There are 1000 classes of different objects that the original VGG16 model was employed to classify. It is not feasible to directly use the original VGG16 mode to classify the four classes of skin images. As a result, the modification must be carried out in accordance with this article. In Fig. 4, the modified VGG16 model is shown.

### Explainable artificial intelligence employing layer-wise relevance propagation

Explainability — the extent to which an AI system can act transparently and comprehensibly, not only apparently but ideally also for all^60^. In other words, this is just about making the process of decision-making understandable and accessible to those end-users who have no technical background. Making DL algorithms’ “black box” decision-making more transparent and intelligible is the goal of explainable artificial intelligence.

The explainability technique used in this article to explain a DL model is LRP. One major technique for explaining networks relying on the back-propagation algorithm is LRP^61^. This study uses the LRP technique for interpretable analysis through whether decisions made by a model reflect meaningful patterns in its input and improve generalization of proposed model. At its core, the LRP algorithm is rooted in exact reversal of contributions to tracking back from final output node layer by layer to single input nodes^62^. Additionally, LRP compensates for the perturbation technique (occlusion map) and the shortcoming of shattered gradients in gradient methods (Grad-CAM)^63^.

## Simulation and results

This article uses Google Colab and Pytorch for simulation and results. Experiment results are measured using several famous statistical metrics from Eqs. (1–8) to evaluate the proposed framework’s classification performance^64^. Skin conditions that are correctly diagnosed are termed true positives (T_p_) or true negatives (T_n_), while skin conditions that are inaccurately diagnosed are termed false positives (F_p_) and false negatives (F_n_). Detailed explanations of the designated statistical metrics are provided below.

### Accuracy

Accuracy is the ratio of correctly predicted instances to the total number of instances in the dataset. It shows how the model will predict the outcome for every possible combination; thus, it is a single measure to evaluate the rate at which the model discriminates one class from another.

1 $$\:\text{A}\text{c}\text{c}\text{u}\text{r}\text{a}\text{c}\text{y}=\frac{Tp+Tn}{Tp+Fp+Fn+Tn}\text{*}100$$

### Misclassification rate

The Misclassification rate is the proportion of instances wrongly classified to the total number of instances. It measures the rate at which a model gives wrong predictions, shedding light on wrongly estimated outcomes.

2 $$\:\text{M}\text{i}\text{s}\text{c}\text{l}\text{a}\text{s}\text{s}\text{i}\text{f}\text{i}\text{c}\text{a}\text{t}\text{i}\text{o}\text{n}\:\text{r}\text{a}\text{t}\text{e}=\frac{Fp+Fn}{Tp+Fp+Fn+Tn}\text{*}100$$

### Precision

Precision is the number of true positives divided by the sum of all false positive and true positive results. It is used to evaluate a model’s ability to accurately predict either label (positive or negative).

3 $$\:\text{P}\text{r}\text{e}\text{c}\text{i}\text{s}\text{i}\text{o}\text{n}=\frac{Tp}{Tp+Fp}\text{*}100$$

### Specificity

Specificity measures the number of instances that are actually negative out of all those predicted as negatives by the model. It would mean that the model classifies all negative instances as belonging to the negative class to prevent false positives.

4 $$\:\text{S}\text{p}\text{e}\text{c}\text{i}\text{f}\text{i}\text{c}\text{i}\text{t}\text{y}=\frac{Tn}{Tn+Fp}\text{*}100$$

### Sensitivity

Sensitivity, also known as a recall or true positive rate (TPR), measures how many of the actual positives are captured by the model. The recall score is essential because it shows how well the model can detect positive cases.

5 $$\:\text{S}\text{e}\text{n}\text{s}\text{i}\text{t}\text{i}\text{v}\text{i}\text{t}\text{y}=\frac{Tp}{Tp+Fn}\text{*}100$$

### False negative rate

FNR measures the number of true positive instances that are classified as false negatives by the model. It shows how frequently the model misclassifies negatives; a high measure here reflects more misses on true positives.

6 $$\:\text{F}\text{N}\text{R}=\frac{Fn}{Fn+Tp}\text{*}100$$

### False positive rate

FPR measures the ratio of actual negative cases predicted positively. It is the proportion of instances that are negative but falsely predicted as positive.

7 $$\:\text{F}\text{P}\text{R}=\frac{Fp}{Fp+Tn}\text{*}100$$

### F1 score

The F1 score, on the other hand, is a metric that considers both precision and sensitivity to have an overall balanced model evaluation in terms of performance under various scenarios where there are many more records per class depending on whether false positives are as important or unimportant as false negatives. The F1 score is calculated using the harmonic mean of precision and sensitivity values, which biases it to lower values.

8 $$\:\text{F}1\:\text{S}\text{c}\text{o}\text{r}\text{e}=\frac{2\text{*}\left(\text{P}\text{r}\text{e}\text{c}\text{i}\text{s}\text{i}\text{o}\text{n}\text{*}\text{S}\text{e}\text{n}\text{s}\text{i}\text{t}\text{i}\text{v}\text{i}\text{t}\text{y}\right)}{\text{P}\text{r}\text{e}\text{c}\text{i}\text{s}\text{i}\text{o}\text{n}+\text{S}\text{e}\text{n}\text{s}\text{i}\text{t}\text{i}\text{v}\text{i}\text{t}\text{y}}$$

These metrics are calculated using a confusion matrix (CM). A CM evaluates the performance of a classification model by breaking down how accurately the model makes predictions about each class. For the proposed model using the modified VGG16, the simulation was set up with a mini-batch size of 32, an optimal epoch count of 10, a learning rate of 0.00001, and the Adam optimization algorithm. The mini-batch size means the model processes 32 samples at a time to calculate gradients and update its parameters. Training the model over different epoch counts showed that 10 epochs gave the best results, with an epoch being a complete pass through the entire training dataset. The Adam optimizer, known for its efficiency and ability to handle noisy data, was used with a learning rate of 0.00001 to ensure smooth and stable training.

**Fig. 5:**
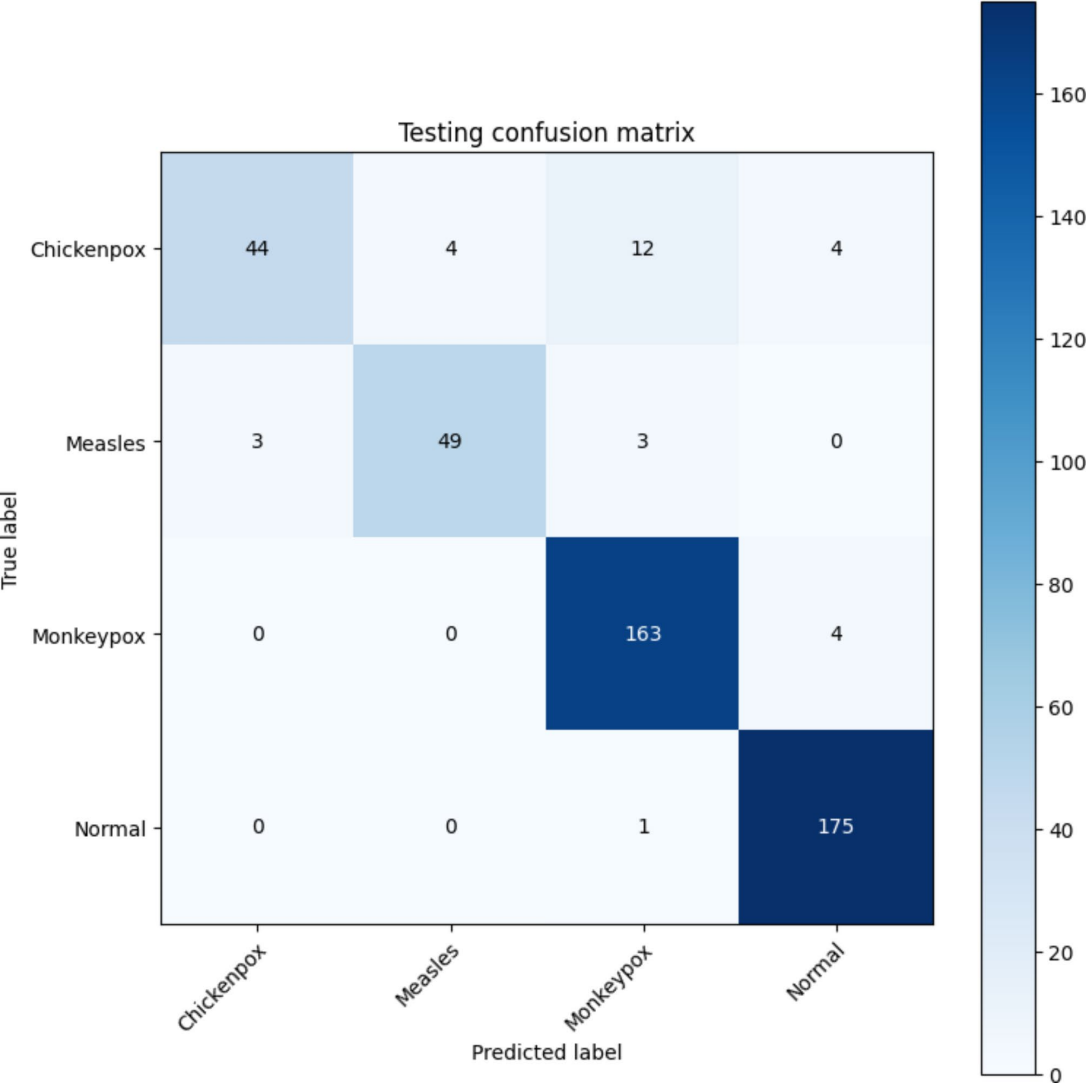
Testing confusion matrix for the proposed model.

Figure 5 displays the testing CM for the proposed model. A total of 462 images were utilized in the 10th epoch. In class chickenpox, 44 out of 64 images were correctly classified, while 20 images were misclassified (4 as measles, 12 as monkeypox, and 2 as normal). In the case of measles, 49 images out of 55 were correctly classified, while 6 images were misclassified (3 images as chickenpox and 3 images as monkeypox). In the case of monkeypox, 163 images out of 167 were correctly classified as monkeypox, while 4 images were misclassified as normal. In the case of class normal, 175 images out of 176 were correctly classified, while only 1 image was misclassified as monkeypox.

Table 3 provides the statistical significance of different performance metrics. It is an extensive assessment of the model’s performance with some evaluation criteria, like correctly predicting instances, minimizing error rate, and balancing trade-offs concerning false negatives vs. true positives.

**Table 3:** Proposed model performance evaluation.

Performance Parameters	Testing
Accuracy	93.29%
Misclassification rate	6.71%
Precision	88.72%
Specificity	97.99%
Sensitivity	93.19%
FNR	6.81%
FPR	2.01%
F1 score	0.91

**Fig. 6:**
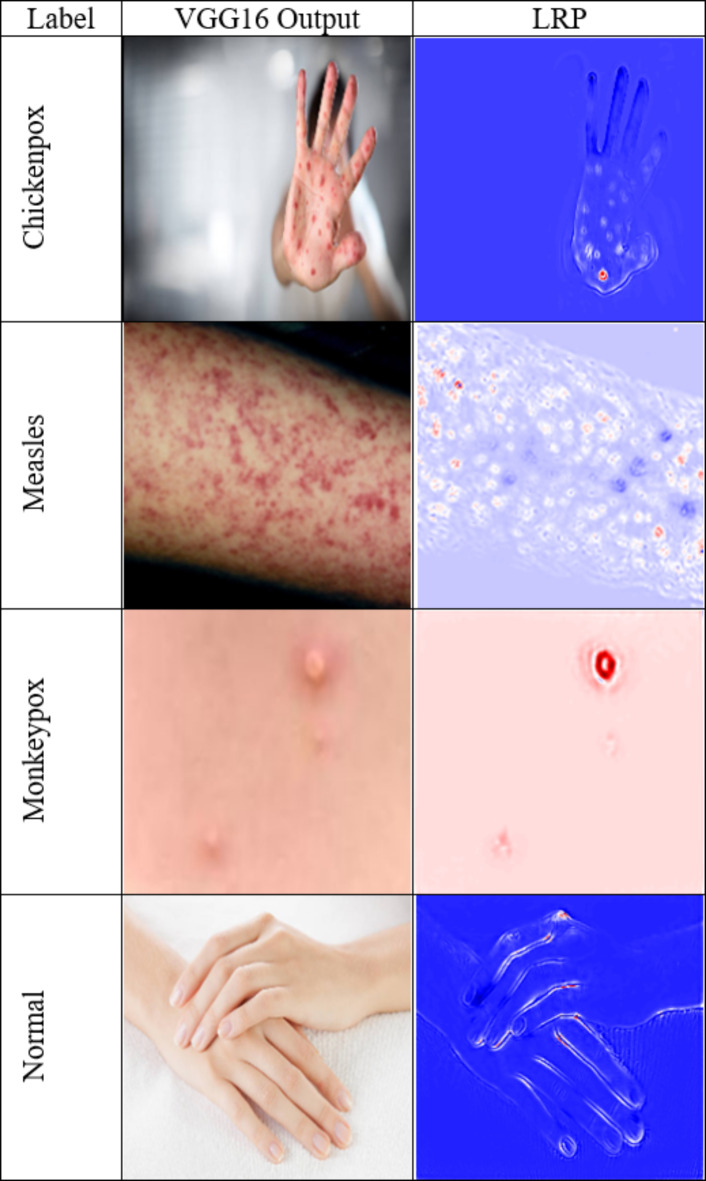
Explanations based on LRP for the VGG16 model.

Figure 6 shows how the LRP approach is applied to show why the VGG16 model gave each particular prediction. LRP is a technique that improves the interpretability of neural networks by backtracking predictions to input features, which thus represents regions in images useful for predicting with each LRP layer. Figure 6 shows the most “important” areas used by VGG16 to distinguish between classes and which regions in classes are marked using LRP. This visual representation confirms the model’s predictive performance and the reasons behind these predictions. For example, suppose the model is picking out specific components of a rash that appear to distinguish it from chickenpox. In that case, it shows how important those pieces are in allowing this image to be categorized correctly.

Several methods have been utilized to identify and classify skin diseases. TL is an innovation for identifying and predicting skin diseases. It uses pre-trained models to improve specific skills. Table 4 compares the proposed model’s performance with other previously reported models. As depicted in Table 4, the proposed model competes and excels over previously reported models with a remarkable accuracy rate of 93.29%. This high accuracy underscores the proposed model’s competitive edge, convincing of its superiority in diagnosing various skin diseases. Furthermore, the proposed model incorporates the XAI technique to increase transparency and fairness.

**Table 4:** Comparison of the proposed model with the literature.

Author	Year	Technique	Use of XAI	Accuracy (%)	Misclassification rate (%)
Ali et al.^47^	2022	VGG16 ResNet50 InceptionV3 Ensemble	No	81.48 ± 6.87 82.96 ± 4.57 74.07 ± 3.78 79.26 ± 1.05	18.52 ± 6.87 17.04 ± 4.57 25.93 ± 3.78 20.74 ± 1.05
Burak Gülmez^48^	2022	Ensemble approach	No	84.2	15.8
Irmak et al.^49^	2022	MobileNetV2 VGG16 VGG19	No	91.38 83.62 78.45	8.62 16.38 21.55
Singh and Songare ^50^	2022	VGG-16 ResNet50 InceptionV3 GoogLeNet	No	83.85 85.38 86.37 88.27	16.15 14.62 13.63 11.73
Sharma et al.^51^	2023	ResNet18-based model	No	84.59	15.41
Sethy et al.^52^	2023	Darknet 19 Improved Darknet 19	No	81.4 85.49	18.6 14.51
Uysal^53^	2023	CNN-LSTM hybrid model	No	87	13
Ariansyah et al.^54^	2023	CNN VGG16	No	64.52 83.33	35.48 16.67
Kundu et al.^55^	2023	SVM KNN RestNet50 ViT	No	65 84 91 93	35 16 9 7
Aqsa Akram et al.^56^	2024	SkinMarkNet	No	90.615	9.385
Proposed model	2024	VGG16 empowered with LRP	Yes	93.29	6.71

## Practical and managerial implications

The proposed integration of VGG16 with LRP offers practical benefits, especially in healthcare. With 93.29% accuracy, it provides a reliable tool for diagnosing skin diseases early and accurately, enhancing patient care and lowering the strain on healthcare systems. By making its predictions explainable, the model builds trust among doctors and patients, addressing the common concern of AI being a ‘black box.’ Automating disease classification can streamline workflows, lower costs, and allow clinics and telemedicine platforms to handle more patients efficiently. It also has the potential to bring accessible diagnostic tools to underserved areas through telehealth applications.

Additionally, the explainability feature aligns with ethical AI practices, helping managers and policymakers ensure transparency, compliance, and confidence in AI-based solutions. This model demonstrates how AI can transform healthcare by combining accuracy, efficiency, and trustworthiness.

## Conclusion and future work

The proposed model, which integrates VGG16 with LRP, achieved a notable accuracy of 93.29% and a misclassification rate of just 6.71%. These results underline the model’s ability to address the limitations of existing methods while incorporating explainability through XAI techniques. LRP enhances transparency by offering meaningful insights into the decision-making process, making it a valuable tool for building trust in AI-based systems.

At the same time, certain limitations were identified. The dataset, while suitable for the scope of this research, may only partially reflect the complexity and diversity of real-world scenarios, potentially limiting the generalizability of the findings. Additionally, privacy concerns related to sensitive data, especially in domains like healthcare, pose significant challenges. Balancing robust privacy-preserving measures with high model performance remains an ongoing priority.

Future efforts could address these limitations by testing the model on larger and more diverse datasets to ensure broader applicability across various domains. Incorporating advanced privacy-preserving approaches like federated learning or blockchain technologies could help mitigate confidentiality concerns. Further exploration of explainability techniques may also enhance the model’s interpretability, making it more accessible and transparent for both technical and non-technical users.

## Data Availability

The dataset & Simulation files used during the current study are available from the corresponding author upon reasonable request.
